# Coexpression of CMTM6 and PD-L1 as a predictor of poor prognosis in macrotrabecular-massive hepatocellular carcinoma

**DOI:** 10.1007/s00262-020-02691-9

**Published:** 2020-08-07

**Authors:** Li-Li Liu, Shi-Wen Zhang, Xue Chao, Chun-Hua Wang, Xia Yang, Xin-Ke Zhang, Yan-lin Wen, Jing-Ping Yun, Rong-Zhen Luo

**Affiliations:** 1 State Key Laboratory of Oncology in South China, Collaborative Innovation Center for Cancer Medicine, Sun Yat-Sen University Cancer Center, Guangzhou, 510060 China; 2 Department of Pathology, Sun Yat-Sen University Cancer Center, 651# Dong Feng Road East, Guangzhou, 510060 Guangdong China; 3 Department of Pathology, The Eighth Affiliated Hospital, Sun Yat-Sen University, Shenzhen, 51800 China

**Keywords:** CMTM6, PD-L1, Inflammatory cells, HCC, Prognosis

## Abstract

The “macrotrabecular-massive” (MTM) pattern of hepatocellular carcinoma (HCC) has been suggested to represent a distinct HCC subtype and is associated with specific molecular features. Since the immune microenvironment is heterogenous in HCC, it is important to evaluate the immune microenvironment of this novel variant. CMTM6, a key regulator of PD-L1, is an important immunocheckpoint inhibitor. This study aimed to evaluate the prognostic effect of CMTM6/PD-L1 coexpression and its relationship with inflammatory cells in HCC. We analyzed 619 HCC patients and tumors were classified into MTM and non-MTM HCC subtypes. The expression levels of CMTM6 and PD-L1 in tumor and inflammatory cells were evaluated by immunohistochemistry. The density of inflammatory cells in the cancer cell nest was calculated. Tumoral PD-L1 expression and inflammatory cell density were higher in the MTM type than in the non-MTM type. CMTM6-high expression was significantly associated with shorter OS and DFS than CMTM6-low expression in the whole HCC patient population and the MTM HCC patient population. Moreover, MTM HCC patients with CMTM6/PD-L1 coexpression experienced a higher risk of HCC progression and death. In addition, CMTM6/PD-L1 coexpression was shown to be related to a high density of inflammatory cells. Notably, a new immune classification, based on CMTM6/PD-L1 coexpression and inflammatory cells, successfully stratified OS and DFS in MTM HCC. CMTM6/PD-L1 coexpression has an adverse effect on the prognosis of HCC patients, especially MTM HCC patients. Our study provides evidence for the combination of immune status assessment with anti-CMTM6 and anti-PD-L1 therapy in MTM HCC patients.

**Electronic supplementary material:**

The online version of this article (10.1007/s00262-020-02691-9) contains supplementary material, which is available to authorized users.

## Introduction

Malignant primary liver tumors are the second leading cause of cancer-related death worldwide, with an increasing incidence in almost all countries [1]. Hepatocellular carcinoma (HCC) is about 85–90% of primary liver cancer and its main risk factors are hepatitis B (HBV) or C (HCV) infection, alcohol consumption and metabolic syndrome [2, 3]. Early HCC can be treated by local ablation, surgical resection or liver transplantation. Kinase and immunocheckpoint inhibitors have been shown to be effective options for the treatment of advanced HCC [4]. Among HCC subtypes, the trabecular pattern is the most common growth pattern of HCC and mimics normal hepatic cord plates. When the trabeculae become > 6 cells thick, the growth pattern is referred to as “macrotrabecular-massive” (MTM), and when it is > 50% of the entire tumor, it is considered a subtype: MTM-HCC [5, 6]. Recent studies have shown that MTM-HCC has an aggressive phenotype and distinct biological (high alpha-feto protein serum levels) and molecular (G3 transcriptomic subgroup, TP53 mutations and FGF19 amplifications) features [5]. There is a critical clinical need to explore the immune microenvironment of this novel HCC subtype.

Blockade of the PD-L1/PD-1 interaction with monoclonal antibodies represents a milestone for anticancer immunotherapy. Indeed, agents targeting PD-1/PD-L1 were recently reported to induce impressive antitumor effects in HCC [7]. Patients with positive PD-L1 expression had significantly poorer DFS and OS than PD-L1 negative patients [8]. Blocking CSF1/CSFR1 prevents tumor-associated macrophage trafficking and is also associated with high responsiveness to PD-1 blockade [9]. Notably, intratumoral heterogeneity of PD-L1 expression has been frequently observed in HCC [10]. Tumors not expressing detectable levels of PD-L1 can also respond to PD-1 inhibitors. Therefore, another predictor to supplement PD-L1 is needed.

CMTM6 belongs to the chemokine-like factor (CKLF)-like MARVEL transmembrane domain-containing (CMTM) family and is broadly expressed at the plasma membrane of various cells, but the biological function of this ubiquitously expressed protein was unknown until recently [11]. MARVEL domain proteins have been suggested as key regulators of PD-L1 in tumor cells [12]. CMTM6 promotes PD-L1 expression in tumor cells in the defense against T cells [13]. In contrast, the depletion of CMTM6 relieves T cell immunosuppression [14]. Additionally, it has been reported that elevated CMTM6 in head and neck squamous cell carcinoma and glioma is associated with a poor prognosis [12, 15], and a potential therapeutic target for renal clear cell carcinoma [16]. CMTM6 expression in combination with PD-L1 expression can be used as a prognostic and therapeutic indicator in lung cancer and pancreatic ductal adenocarcinoma [17–20]. Previous studies found that CMTM6 was downregulated in HCC tissues and correlated with HCC metastasis and survival in HCC patients, the polymorphisms of rs164207 in CMTM6 was found in HCC [21–23]. In addition, CMTM6 showed decreased expression in nonneoplastic liver cells after tumor promotion with piperonyl butoxide (PBO) in mice [24]. These findings suggest the potential value of CMTM6 as a therapeutic target. However, the association between CMTM6 and the immune microenvironment has not been evaluated in HCC subtypes.

In our study, we explored the relationship between CMTM6 expression, clinicopathological variables, and the immune microenvironment by analyzing data from the whole population and two histological subtypes. Then, based on CMTM6 expression, PD-L1 expression and inflammatory cell density, we optimized the present immune classification and established a novel immunophenotyping system in HCC, especially in MTM-HCC. Our study may provide evidence for HCC patients to choose proper immunotherapy.

## Materials and methods

### Patients and samples

Our study was approved by the Institutional Ethical Boards of Sun Yat-sen University Cancer Center. Patient data were retrospectively analyzed from two subtypes: the MTM type (316 patients) and the non-MTM type (303 patients). Patients in both subtypes underwent surgical resection for HCC from Jan. 2000 to Dec. 2010. Informed consent was obtained from all patients. The mean follow-up time was 32.3 months, and the sample included 549 (88.7%) males and 70 (11.3%) females. The mean age was 49 years, ranging from 13 to 77 years.

### Tissue microarray (TMA) construction

HCC tissues and adjacent nontumorous hepatic tissue samples were collected and constructed for TMA. Primary antibodies (anti-CMTM6: Sigma-Aldrich, HPA 026980; anti-PD-L1: Roche, SP263) were incubated at 4 °C, washed three times with phosphate-buffered saline, incubated with biotinylated goat anti-mouse antibodies, and then stained with DAKO liquid 3,3′-diaminobenzidine tetrahydrochloride (DAB) and finally with Mayer’s hematoxylin. TMA slides stained with CMTM6 and PD-L1 were observed under a microscope, and the protein expression levels of CMTM6 and PD-L1 were assessed by two independent pathologists (Shi-Wen Zhang and Xue Chao).

The CMTM6 positively stained samples were scored as follows: 0, less than 5% positively stained cells; 1, 6–19% positively stained cells; 2, 20–49% positively stained cells; 3, 50–74% positively stained cells; 4, 75–100% positively stained cells. The intensity was scored as follows: 0, negative staining; 1, weak staining; 2, moderate staining; and 3, strong staining. The final score was calculated by multiplying the percentage score by the staining intensity score. The median IHC score of 3 was chosen as the cut-off value for defining high and low CMTM6 expression. For tumor PD-L1 expression, the percentages of cells demonstrating membranous staining for PD-L1 among total tumor cells were quantified, consistent with previous studies [25, 26]. For inflammatory cell PD-L1 expression, any expression (≥ 1%) of PD-L1 on tumor infiltrating and stromal immune cells was considered present. The tumor and inflammatory cell PD-L1 positivity threshold was defined as at least 1% displaying membranous PD-L1 staining of any intensity. The density of inflammatory cells was manually counted in five separate fields under × 200 high-power magnification. The inflammatory cell positivity threshold was defined as at least 1/mm^2^. Quantification was conducted independently by two experienced pathologists who were blinded to the clinical data of patients, any discrepancies in scoring were adjudicated.

### Statistical analysis

SPSS 19.0 was used to perform statistical analyses (SPSS, Chicago, IL, USA). Student’s *t* test was used to assess the significance of differences in CMTM6 and PD-L1 expression and inflammatory cell density levels. The Chi-square test was used to analyze the correlation between CMTM6 expression and clinicopathological parameters in HCC patients. Pearson’s *χ*2 test was used to analyze the correlation between the expression of CMTM6 and PD-L1. OS and DFS were analyzed by Kaplan–Meier analysis and compared by log-rank test. Univariate and multivariate Cox regression analyses were used to analyze prognostic correlations. *P* < 0.05 was considered statistically significant.

## Results

### Patient baseline characteristics according to tumor histological subtype

**Fig. 1:**
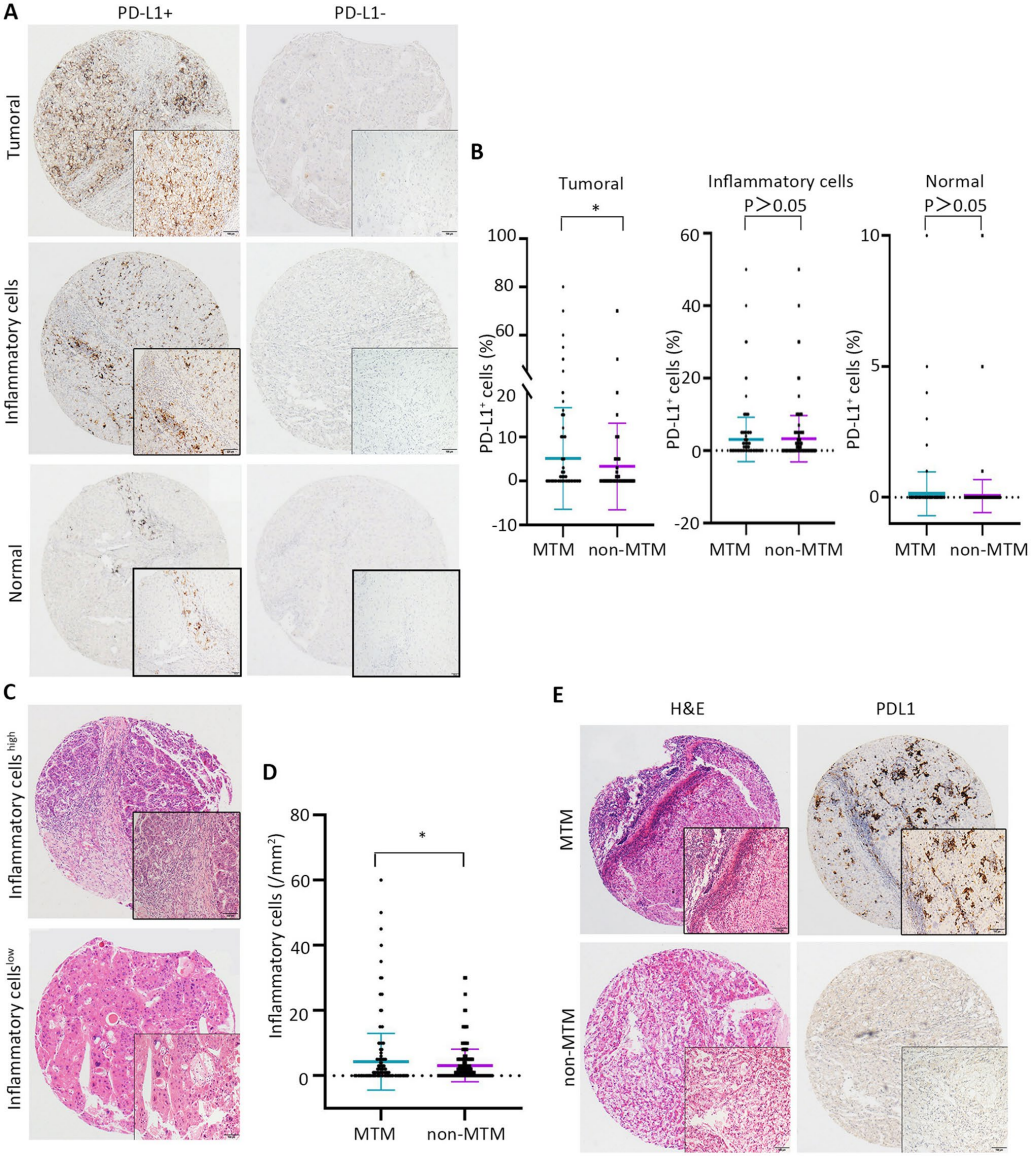
PD-L1 expression and inflammatory cell density in MTM subtype and non-MTM subtype of HCC. **a** Expression of PD-L1 in HCC detected by IHC. Tumoral PD-L1-positive and -negative tumors (upper panel), inflammatory cell PD-L1-positive and -negative tumors (middle panel) and adjacent normal cell PD-L1-positive and -negative expression (lower panel). **b** Dot plots of tumoral, inflammatory cell and normal cell PD-L1-positive percentage in MTM type vs. non-MTM type tumors. *P* values were calculated using the Mann–Whitney *U* test. **c** Representative images of high and low inflammatory cell density in MTM type and non-MTM type tumors. **d** Dot plot of inflammatory cells in MTM type vs. non-MTM type tumors. **e** Representative HE and IHC images of high inflammatory cells density and tumoral PD-L1-positve in MTM type (upper panel) and low inflammatory cell density and tumoral PD-L1-negative in non-MTM type (lower panel). Quantitative data are presented as mean ± SD

**Fig. 2:**
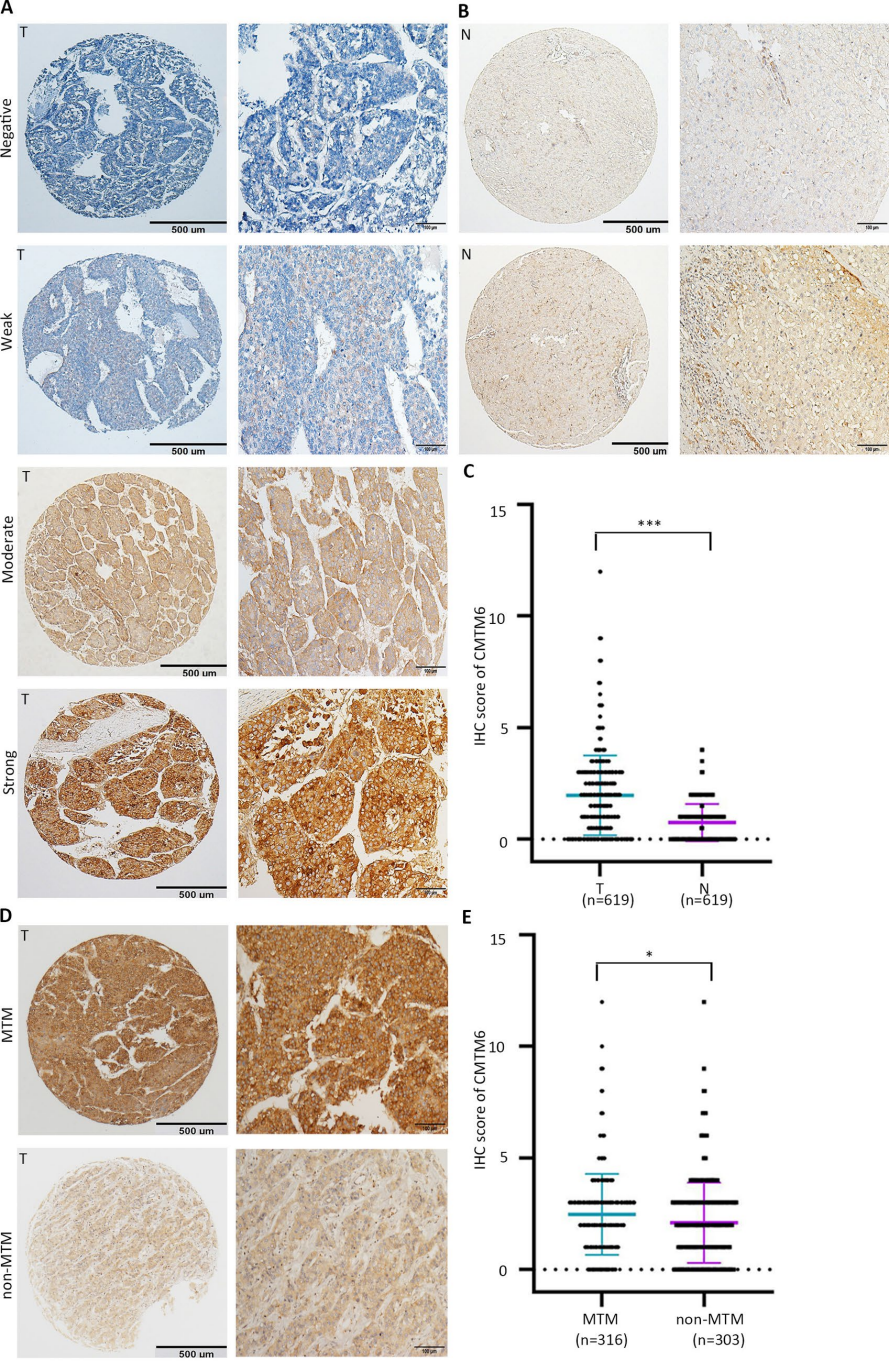
Overexpression of CMTM6 in HCC detected by IHC. **a** Representative images of IHC staining for CMTM6 expression in a TMA cohort. Representative images of negative, weak, moderate and strong (top to bottom) density staining for tumor tissues are shown. **b** Representative IHC images of negative (top) and positive (bottom) in non-tumor tissues are presented. **c** The IHC scores of the TMA cohort, including 619 HCC patients. *P* values were calculated using the Student’s *t *test. **d** Representative images of weak and strong density staining in MTM type and non-MTM type tumors. **e** Dot plots of IHC score in MTM type vs. non-MTM type tumors. Quantitative data are presented as mean ± SD

A total of 619 HCC patients who underwent primary tumor resection were analyzed in this study. The clinicopathological variables of patients with the two histological subtypes of HCC are listed in Supplementary Table 1. Representative IHC images for CMTM6 and PD-L1 in HCC are shown in Figs. 1 and 2. In both histological subtypes, the expression rates of CMTM6 expression were high, at 63.9% (202/316) and 52.8% (160/303); interestingly enough, these rates were significantly higher than the rates of tumoral PD-L1 expression, which were 43.0% (136/316) and 34.0% (103/303) (Supplementary Table 1). Consistent with previous studies, our results showed that MTM type has a worse prognosis than non-MTM type (Supplementary Fig. 1a, b).

### PD-L1 expression, inflammatory cell density and CMTM6 expression in HCC tissues

We examined PD-L1 expression in tumor tissues and found that 38.6% (239/619) of patients were positive for tumoral PD-L1, 50.1% (310/619) of patients were positive for inflammatory cell PD-L1 and 2.4% (15/619) of patients were positive for adjacent normal cell PD-L1 (Fig. 1a; Supplementary Table 1). The percentage of PD-L1+ tumor cells was higher in MTM-type tumors than in non-MTM type tumors (Fig. 1b). The density of inflammatory cells in the tumoral region was significantly higher in MTM type tumors than in non-MTM type tumors (Fig. 1c, d). However, inflammatory cell PD-L1 and normal cell PD-L1 expression was not significantly different between the MTM type and non-MTM type (Fig. 1b). Representative photomicrographs are shown in Fig. 1e.

We next further confirmed the expression profile of CMTM6 in HCC. Representative IHC images of CMTM6 expression are shown in Fig. 2a. CMTM6 expression in HCC was significantly higher than that in nontumorous tissues (Fig. 2b, c). The expression of CMTM6 was significantly higher in MTM-type tumors than in non-MTM-type tumors (Fig. 2d, e). In another TMA cohort consisting of 47 HCC cases with portal vein embolus, CMTM6 expression was not significantly different between primary lesions and tumor embolus metastases (Supplementary Fig. 1c, d).

### Relationship of CMTM6 and PD-L1 expression and clinicopathological features

The association between CMTM6 expression and clinicopathological characteristics is shown in Table 1. CMTM6 expression was significantly correlated with high AFP level (*P* = 0.032), tumor size (*P* = 0.016), advanced TNM stage (*P* < 0.001), vascular invasion (*P* = 0.001), and lymph node metastasis (*P* = 0.030) in the MTM type but not in the non-MTM type. This significant association was also detected between CMTM6 and tumoral PD-L1 expression in both subtypes (MTM type: *P* < 0.001; non-MTM type: *P* = 0.004).

**Table 1:** The association of clinicopathological parameters with CMTM6 expression in the MTM and non-MTM HCC

Variables	Overall (*n* = 619)				MTM (*n* = 316)				Non-MTM (*n* = 303)			
	*n*	CMTM6 expression			*n*	CMTM6 expression			*n*	CMTM6 expression		
		Low	High	*P* value		Low	High	*P* value		Low	High	*P* value
Age (years)^a^				0.722				0.816				0.230
< 50	291	123	168		166	65	101		125	58	67	
≥ 50	328	134	194		150	49	101		178	85	93	
Gender				**0.005**				0.188				**0.004**
Male	549	217	332		273	90	183		276	127	149	
Female	70	40	30		43	24	19		27	16	11	
HBV				0.641				0.950				0.463
Positive	47	18	29		24	7	17		23	11	12	
Negative	572	239	333		292	107	185		280	132	148	
AFP (ng/ml)				**0.012**				**0.032**				0.374
< 20	149	75	74		61	25	36		88	50	38	
≥ 20	470	182	288		255	89	166		215	93	122	
Cirrhosis				0.557				0.537				0.939
Yes	109	48	61		52	19	33		57	29	28	
No	510	209	301		264	95	169		246	114	132	
Tumor multiplicity				0.087				0.053				0.758
Single	427	187	240		210	77	133		217	110	107	
Multiple	192	70	122		106	37	69		86	33	53	
Tumor size^b^				**0.003**				**0.016**				0.181
< 5 cm	168	86	82		70	30	40		98	56	42	
≥ 5 cm	451	171	280		246	84	162		205	87	118	
Differentiation				0.098				0.111				0.842
Well	58	30	28		21	8	13		37	22	15	
Moderate–poor	561	227	334		295	106	189		266	121	145	
TNM stage				**0.001**				**0.000**				0.567
I–II	377	176	201		179	67	112		198	109	89	
III–IV	242	81	161		137	47	90		105	34	71	
Vascular invasion				**0.002**				**0.001**				0.418
No	521	230	291		250	93	157		271	137	134	
Yes	98	27	71		66	21	45		32	6	26	
Tumor capsule				0.151				0.358				0.396
Complete	351	137	214		190	65	125		161	72	89	
Incomplete	268	120	148		126	49	77		142	71	71	
LN metastasis				0.226				**0.030**				0.653
No	587	247	340		299	107	192		288	140	148	
Yes	32	10	22		17	7	10		15	3	12	
Cytological type				0.111				0.124				0.447
Liver cell	538	231	307		277	102	175		261	129	132	
Clear cell	40	16	24		19	8	11		21	8	13	
Fatty-rich	33	9	24		16	3	13		17	6	11	
Giant cell	8	1	7		4	1	3		4	0	4	
Tumoral PD-L1				**0.000**				**0.000**				**0.004**
Negative	380	188	192		180	77	103		200	111	89	
Positive	239	69	170		136	37	99		103	32	71	
Inflammatory cell PD-L1				**0.000**				**0.000**				0.093
Negative	309	152	157		152	62	90		157	90	67	
Positive	310	105	205		164	52	112		146	53	93	
Inflammatory cells				**0.000**				**0.012**				**0.007**
Low	264	133	131		124	56	68		140	77	63	
High	355	124	231		192	58	134		163	66	97	

*AFP* a-fetoprotein, *HBV* hepatitis B virus infection, *LN* lymph node

*P value < 0.05 in bold are statistically significant

^a^Median age; ^b^Median tumor size

The association between tumoral PD-L1 expression and clinicopathological variables is shown in Supplementary Table 2. Tumor PD-L1 positivity was significantly correlated with age (*P* = 0.032), HBV positivity (*P* = 0.045), and high AFP level (*P* = 0.008) in the MTM type but not in the non-MTM type. A significant association was identified between tumoral PD-L1 positivity and high inflammatory cell density in both subtypes (MTM type: *P* < 0.001; non-MTM type: *P* < 0.001).

### Relationship between CMTM6/PD-L1 coexpression and inflammatory cells in HCC

**Fig. 3:**
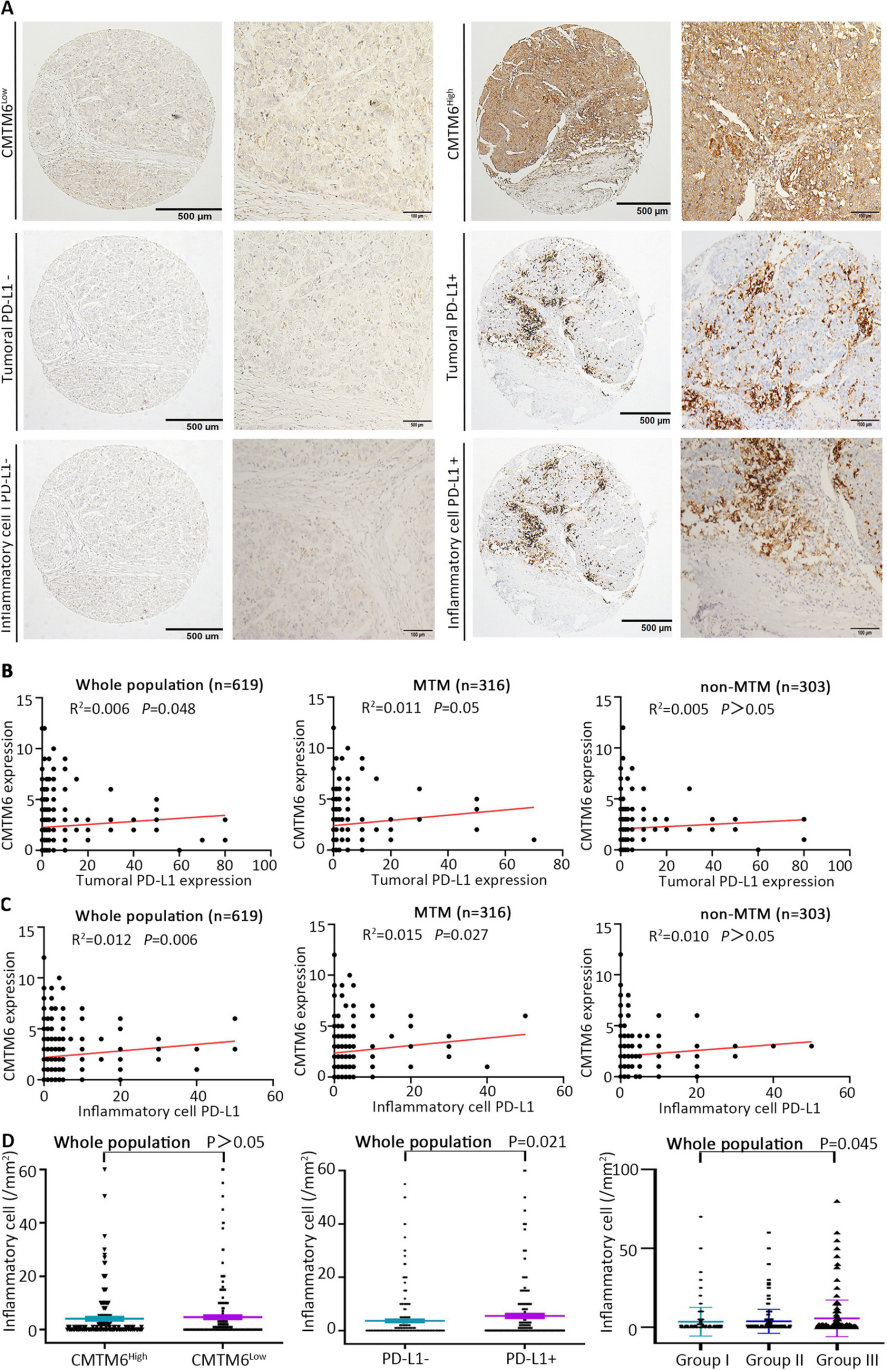
PD-L1 and CMTM6 expression in HCC tissue. **a** Representative micrographs of CMTM6-high and -low, tumoral PD-L1-positive and -negative, inflammatory cell PD-L1-positive and -negative expression. **b** Scatter plot correlation between CMTM6 and tumoral PD-L1 expression in whole population, MTM type and non-MTM type. **c** Scatter plot correlation between CMTM6 and inflammatory cell PD-L1 expression in whole population, MTM type and non-MTM type. **d** Dot plots of inflammatory cell density in different subgroups of whole population. Group I: CMTM6Low/PD-L1−; Group II: CMTM6High/PD-L1− or CMTM6Low/PD-L1+; Group III: CMTM6High/PD-L1+. Quantitative data are presented as mean ± SD. *PD-L1−* PD-L1-negative, *PD-L1+* PD-L1-positive

We also detected the density of inflammatory cells in HCC. Representative figures are shown in Fig. 3a. An association was found between high CMTM6 expression and tumoral PD-L1 in the whole population (*R*^2^ = 0.006, *P* = 0.048) and in MTM-type HCC (*R*^2^ = 0.006, *P* = 0.050), but no significant association was found in non-MTM-type HCC (Fig. 3b). A positive association was detected between high CMTM6 expression and inflammatory cell PD-L1 in the whole population (*P* = 0.006) and MTM type (*P* = 0.027) (Fig. 3c). As shown in Fig. 3d, no significant difference was found between CMTM6-high and CMTM6-low tumors. The density of inflammatory cells in the PD-L1+ tumors was significantly higher than that in PD-L1− tumors in the whole population (*P* = 0.021). Furthermore, the density of inflammatory cells was highest in group III (CMTM6-high/PD-L1+) in the whole population (*P* = 0.045). In MTM type and non-MTM type, no significant difference among different subgroup were detected (Supplementary Fig. 1e, f).

### Impact of CMTM6 and PD-L1 expression on the overall survival and disease-free survival of HCC patients

To explore the prognostic significance of CMTM6 and PD-L1, Cox proportional hazards regression was performed. Among the whole population, CMTM6-high patients had significantly increased risks of disease progression and all causes of death compared with CMTM6-low patients (OS: HR = 1.493, *P* < 0.001; DFS: HR = 1.369, *P* = 0.010) and MTM type patients (OS: HR = 1.574, *P* < 0.001; DFS: HR = 1.624, *P* = 0.008). In the non-MTM type, patients with high CMTM6 tended to have a poor prognosis (OS: HR = 1.310, *P* = 0.035), but there was no association with DFS (Table 2; Supplementary Table 3).

**Table 2:** Univariate analyses of prognostic factors correlated with OS in MTM and non-MTM HCC

Variables	Overall		MTM		Non-MTM	
	HR (95% CI)	*P* value	HR (95% CI)	*P* value	HR (95% CI)	*P* value
Overall survival						
Age (years)^a^ (< 50 vs. ≥ 50)	0.886 (0.746–1.053)	0.169	0.828 (0.643–1.067)	0.144	0.833 (0.584–1.189)	0.315
Gender (male vs. female)	0.767 (0.578–1.017)	0.066	0.625 (0.391–1.000)	**0.050**	1.006 (0.794–1.276)	0.958
HBV (positive vs. negative)	1.331 (0.948–1.869)	0.099	1.214 (0.741–1.991)	0.441	1.471 (0.922–2.347)	0.106
AFP (ng/ml) (< 20 vs. ≥ 20)	1.227 (1.005–1.498)	**0.045**	1.263 (0.959–1.664)	0.097	1.073 (0.799–1.441)	0.639
Cirrhosis (no vs. yes)	1.064 (0.841–1.346)	0.604	1.110 (0.791–1.558)	0.545	1.003 (0.724–1.390)	0.985
Tumor multiplicity (single vs. multiple)	1.633 (1.359–1.963)	**0.000**	1.771 (1.349–2.324)	**0.000**	1.478 (1.151–1.897)	**0.002**
Tumor size^b^ (cm) (< 5 vs. ≥ 5)	1.618 (1.322–1.980)	**0.000**	1.497 (1.136–1.973)	**0.004**	1.665 (1.232–2.250)	**0.001**
Differentiation (well vs. moderate–poor)	1.535 (1.143–2.062)	**0.004**	1.479 (1.007–2.170)	**0.046**	1.513 (0.949–2.415)	0.082
TNM stage (I–II vs. III–IV)	1.778 (1.491–2.120)	**0.000**	1.874 (1.446–2.429)	**0.000**	1.640 (1.290–2.086)	**0.000**
Vascular invasion (no vs. yes)	2.452 (1.952–3.081)	**0.000**	3.472 (2.362–5.103)	**0.000**	1.901 (1.427–2.532)	**0.000**
Tumor capsule (complete vs. incomplete)	0.746 (0.627–0.889)	**0.001**	0.657 (0.510–0.846)	**0.001**	0.886 (0.696–1.130)	0.330
LN metastasis (no vs. yes)	2.619 (1.817–3.775)	**0.000**	4.290 (2.524–7.292)	**0.000**	1.754 (1.056–2.912)	**0.030**
Cytological type (liver cell vs. clear cell vs. fatty-rich vs. giant cell)	0.983 (0.860–1.124)	0.799	1.062 (0.877–1.285)	0.538	0.897 (0.733–1.096)	0.288
Tumoral PD-L1 (− vs. +)	1.217 (1.021–1.452)	**0.029**	1.610 (1.247–2.102)	**0.000**	0.899 (0.706–1.144)	0.386
Inflammatory cell PD-L1 (− vs. +)	1.161 (0.978–1.379)	0.089	1.458 (1.135–1.875)	**0.003**	0.926 (0.729–1.175)	0.526
Inflammatory cells (low vs. high)	1.038 (0.872–1.235)	0.676	1.198 (0.931–1.542)	0.160	0.855 (0.671–1.089)	0.206
CMTM6 expression (low vs. high)	1.493 (1.250–1.783)	**0.000**	1.574 (1.221–2.028)	**0.000**	1.310 (1.019–1.684)	**0.035**

*AFP* a-fetoprotein, *HBV* hepatitis B virus infection, *LN* lymph node, *HR* hazard ratio, *CI* confidence interval

*P value < 0.05 in bold are statistically significant

^a^Median age; ^b^Median tumor size

**Fig. 4:**
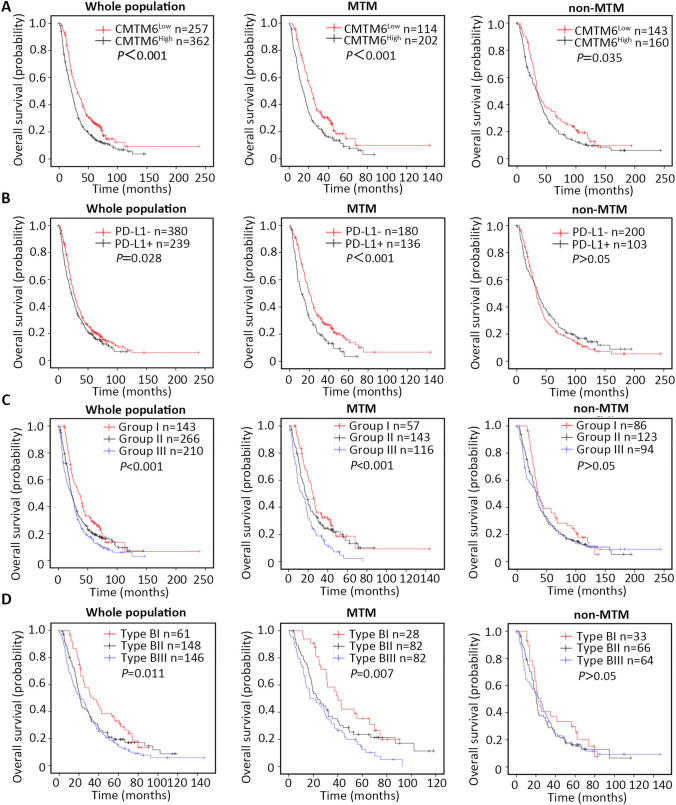
Kaplan–Meier survival curves for OS of HCC patients according to CMTM6 and PD-L1 expression. **a** OS according to CMTM6 expression status in the whole population, MTM and non-MTM type HCC. **b** OS according to tumoral PD-L1 expression status in the whole population, MTM and non-MTM type HCC. **c** OS according to a combination of CMTM6/PDL1 coexpression. Group I: CMTM6Low/PD-L1−; Group II: CMTM6High/PD-L1− or CMTM6Low/PD-L1+; Group III: CMTM6High/PD-L1+. **d** OS according to new immune classification. Type BI: inflammatory cells positive and both negative (CMTM6Low/PD-L1−); type BII: inflammatory cells positive and single positive (CMTM6High/PD-L1− or CMTM6Low/PD-L1+); type BIII: inflammatory cells positive and both positive (CMTM6High/PD-L1+). *PD-L1−* PD-L1-negative, *PD-L1+* PD-L1-positive

Kaplan–Meier analysis revealed that CMTM6 expression was significantly associated with worse DFS and OS in the whole population (OS: *P* < 0.001; DFS: *P* = 0.010) and in the MTM type (OS: *P* < 0.001; DFS: *P* = 0.008) but not in the non-MTM type (Fig. 4a; Supplementary Fig. 2a). Stratified survival analysis further confirmed the prognostic value of CMTM6 (Supplementary Fig. 3; Table 1). Multivariate analysis indicated that high CMTM6 expression was an independent prognostic marker for HCC in whole population (HR = 1.056, *P* = 0.030), but not in the MTM type and non-MTM type (Table 3).

**Table 3:** Multivariate analyses of prognostic factors correlated with OS in MTM and non-MTM HCC

Variables	Whole population		MTM		Non-MTM	
	HR (95% CI)	*P* value	HR (95% CI)	*P* value	HR (95% CI)	*P* value
Overall survival						
Gender (male vs. female)			0.616 (0.379–1.000)	**0.050**		
AFP (ng/ml) (< 20 vs. ≥ 20)	1.016 (0.825–1.251)	0.883				
Tumor multiplicity (single vs. multiple)	1.361 (1.110–1.669)	**0.003**	1.672 (1.232–2.269)	**0.001**	1.197 (0.906–1.582)	0.205
Tumor size (cm) (< 5 vs. ≥ 5)	1.414 (1.127–1.773)	**0.003**	1.427 (1.020–1.996)	**0.038**	1.446 (1.041–2.008)	**0.028**
Differentiation (well vs. moderate–poor)	1.275 (0.939–1.730)	0.119	1.298 (0.868–1.941)	0.204		
TNM stage (I–II vs. III–IV)	1.114 (0.888–1.398)	0.350	1.061 (0.743–1.514)	0.747	1.163 (0.861–1.572)	0.325
Vascular invasion (no vs. yes)	1.742 (1.347–2.253)	**0.000**	2.246 (1.452–3.474)	**0.000**	1.557 (1.130–2.128)	**0.005**
Tumor capsule (complete vs. incomplete)	0.916 (0.759–1.105)	0.359	0.785 (0.597–1.032)	0.083		
LN metastasis (no vs. yes)	2.032 (1.384–2.984)	**0.000**	4.049 (2.328–7.044)	**0.000**	1.446 (0.827–2.425)	0.205
Tumoral PD-L1 (− vs. +)	1.167 (0.973–1.400)	0.097	1.384 (0.994–1.928)	0.054		
Inflammatory cell PD-L1 (− vs. +)			1.384 (0.883–1.676)	0.231		
CMTM6 expression (low vs. high)	1.056 (1.005–1.109)	**0.030**	1.116 (0.850–1.464)	0.430	1.280 (0.994–1.647)	0.055

*AFP* a-fetoprotein, *HBV* hepatitis B virus infection, *LN* lymph node, *HR* hazard ratio, *CI* confidence interval

*P value < 0.05 in bold are statistically significant

^a^Median age; ^b^Median tumor size

Similar to the patterns observed with CMTM6, tumoral PD-L1+ patients suffered much higher mortality rates than PD-L1− patients in the whole population and the MTM type (HR = 1.217, *P* = 0.029 in the whole population; HR = 1.610, *P* < 0.001 in the MTM type) but not in the non-MTM type population (Table 2). Kaplan–Meier analysis also suggested that PD-L1 positivity was significantly associated with worse OS in the whole population and the MTM type (Fig. 4b, whole population, *P* = 0.028; MTM type, *P* < 0.001) but was not associated with worse OS in the non-MTM type. PD-L1 expression was not significantly associated with worse DFS in the respective groups (Supplementary Fig. 2b).

### Coexpression of CMTM6 and PD-L1 in HCC and prognostic significance

Considering that CMTM6 has a regulator function on PD-L1, we attempted to explore the prognostic impact of CMTM6/PD-L1 coexpression in HCC. The proportions of patients with CMTM6-high/PD-L1+, CMTM6-high/PD-L1− or CMTM6-low/PD-L1+, and CMTM6-low/PD-L1− expression are shown in Supplementary Table 1. Patients were divided into three groups: group I, both negative (CMTM6-high/PD-L1−); group II, single positive (CMTM6-high/PD-L1− or CMTM6-high/PD-L1+; and group III, both positive (CMTM6-high/PD-L1+).

Kaplan–Meier analysis demonstrated that both OS and DFS in group III were significantly reduced compared with those in group I and group II in the whole population and those in the MTM type, but not in the non-MTM type (Fig. 4c; Supplementary Fig. 2c). Importantly, multivariate analysis revealed that CMTM6-high/PD-L1+ still had a significant impact on OS in the whole population (HR = 1.213, *P* = 0.005) and MTM type (HR = 1.297, *P* = 0.003) but not in the non-MTM type (Supplementary Table 4).

### Immune classification for HCC

We classified the patients into six types based on inflammatory cell and CMTM6/PD-L1 coexpression to provide rationale for immunotherapy. As shown in Fig. 4d and Supplementary Fig. 2d, in the inflammatory cell positive subgroups, Kaplan–Meier analysis demonstrated that both OS and DFS in type BIII were significantly reduced compared with type BI and type BII in the whole population and the MTM type, but there was no significant difference in the non-MTM type. Furthermore, in the inflammatory cell-negative subgroup, OS in type AIII was significantly reduced compared with that in type AI and type AII in the whole population and the MTM type, but this difference was not observed in the non-MTM type (Supplementary Fig. 1g). However, no significant difference in DFS was detected among the three groups (Supplementary Fig. 2e).

## Discussion

The immunological properties of human malignancies can vary greatly according to tumor origin and histological type and often display diverse immune cell recruitment. This study is the first to reveal remarkable differences in CMTM6 and PD-L1 expression and inflammatory cell density between the two subtypes of HCC, which are intimately related to their tumor biology and clinical outcomes. We found significantly high tumoral PD-L1 expression, high CMTM6 expression and high inflammatory cell density in the MTM type, whereas the non-MTM type showed low inflammatory cell density. CMTM6/PD-L1 expression was an independent prognostic factor for patient survival in the whole population and the MTM subtype population, confirming the crucial roles of these markers in the pathogenesis of HCC. In addition, there was a significant association between CMTM6 and tumoral PD-L1 expression in these two subtypes but no significant association between CMTM6 and inflammatory cell PD-L1 expression in non-MTM subtypes, suggesting that high CMTM6 activity in HCC contributes to tumoral PD-L1 expression. Collectively, these findings provide an important clue for deciphering the distinct clinical features of the two subtypes in patients with HCC.

Recent studies have shown that CMTM6 combined with its family member CMTM4 can maintain the stability of PD-L1 and prevent PD-L1 from lysosome hydrolyzation in multiple tumor types, such as melanoma, breast cancer, and lung cancer [13, 14]. Zhu et al. showed that CMTM6 was downregulated in HCC tissues, supporting a tumor suppressive role of CMTM6 in HCC [21]. However, according to our data, CMTM6 expression was up-regulated in HCC tissues. Elevated expression of CMTM6 was frequently accompanied with worse malignant phenomenon, such as with high AFP level, large tumor size, advanced TNM stage, vascular invasion in a large cohort of 619 HCC cases (In Zhu’s study, only 75 HCC samples were collected). Our data was in line with other studies indicating that increased CMTM6 expression was presented in gliomaand Head and Neck Squamous Cell Carcinoma, and CMTM6 positivity was associated with shorter DFS and OS [12, 15]. Collectively, we consider our data are more representative to show the expression of CMTM6 in HCC. Moreover, high expression of CMTM6 was an independent prognostic factor in the whole population. Expression of CMTM6 was more frequent than expression of PD-L1, consistent with prior literature [18, 19], which indicates that CMTM6 could be regarded as a potential target for immunotherapy. Consistent with our results, upregulation of PD-L1 is observed during HBV infection [27]. Recent studies have shown that extracellular vesicles (EVs) produced by HBV-infected hepatocytes are endocytosed by circulating monocytes resulting in PD-L1 upregulation [28, 29]. Interestingly, another study showed that serum soluble PD-L1 (sPD-L1) concentration was several-fold higher in HBV-related HCC than in healthy control, a significant difference, while sPDL1 was positively correlated with tumor PD-L1 expression [30]. During chronic HBV infection, virus particles are continuously released from virus-infected cells and maintain a network of immunosuppressive mechanisms that interfere with virus elimination [31]. That may explain the reason why PD-L1 expression correlated with HBV infection. We are also very interested in why age is related to PD-L1 expression. However, when we used the Student’s *t* test to analyze the difference in the mean age between the PD-L1 positive group and the negative group, we did not observe with a significant difference (48.62 years vs 50.38 years, *P* = 0.078). It may need more data to support to concluded that PD-L1 is age-related.

Although the expression of both CMTM6 and PD-L1 is induced by related immunoregulatory factors, a previous report indicated that in advanced-stage non-small-cell lung cancer, the proportion of CMTM6-high/PD-L1+ cells is low, and some cases with PD-L1− contain high expression of CMTM6 [18]. CMTM6 was more prevalent than PD-L1 and CMTM6 overexpression was common [19]. Similarly, in our results, we observed that the percentage of CMTM6-high/PD-L1+ cells was only 36.7% (116/316) in the MTM type and 31.0% (94/303) in the non-MTM type. To the best of our knowledge, our study is the first to investigate the clinical significance of CMTM6 and PD-L1 coexpression in HCC. We found that CMTM6/PD-L1 can be regarded as a predictor of OS in HCC patients, especially in patients with the MTM subtype. Our study provides some useful insights for immunotherapy of MTM subtypes: patients with CMTM6-high/PD-L1+ status may benefit from the dual blockade of PD-L1 and CMTM6; patients with one positive immune marker (PD-L1 or CMTM6) may need corresponding immune blockage to improve efficacy; for patients with no positive immune maker, immunotherapy targeting other markers, such as PD-L2 or CMTM4, may be needed.

The relationship between CMTM6 expression and inflammatory cell infiltration is controversial. Chen et al. found that high expression of CMTM6 was associated with low inflammatory density in head and neck squamous cell carcinoma [15]. Wang et al. observed that there was a significant correlation between CMTM6 expression and inflammatory cells in lung adenocarcinoma [32]. In our study, we observed that high CMTM6 expression was significantly associated with a high density of inflammatory cells in HCC. We hypothesize that CMTM6 may be induced by inflammatory cells. In addition, we found high inflammatory cell density in the MTM subtype. This result suggests that MTM-type patients are suitable candidates for tumor immunotherapy. In contrast, low levels of inflammatory cell density in the non-MTM subtype suggest that this subtype has features of nonimmunogenic “cold” tumors, in accordance with previous reports on the poor immunogenicity of HCC, as defined by the lack of tumor-infiltrating lymphocytes and a poor response to immunotherapy [33]. According to Teng’s theory, it may be much more rational to classify by inflammatory cells via the combination PD-L1 and CMTM6 or in combination with other CMTM family members in HCC [34]. Consistent with previous study, our results demonstrated that in inflammatory cell-positive groups, CMTM6/PD-L1 coexpression displayed significant prognostic value for DFS and OS in MTM subtype [35]. These results suggested that both CMTM6 and PD-L1 perform immunosuppressive functions that partly depend on suppression of the inflammatory cell function.

There are some limitations to our study. First, due to the retrospective nature of our study, some bias was inevitable. Second, we only detected CMTM6 and PD-L1 to conduct immune classification. Other immunosuppressive factors, such as PD-L2, may also contribute to immunosuppression in the tumor microenvironment. Therefore, an immunophenotyping system containing other markers is needed to guide immunotherapy.

## Electronic supplementary material

Below is the link to the electronic supplementary material.

Supplementary file1. **a** Representative photomicrographs are shown for the MTM type and non-MTM type. **b** Kaplan–Meier survival curves for OS in MTM type vs. non-MTM type tumor. **c** CMTM6 expression in 47 HCC metastasis cases analyzed by IHC. Representative photomicrographs are shown for the primary tumor (T) and metastatic (M) lesions. **d** Comparison of CMTM6 levels between the primary tumor and metastatic lesions. **e** OS according to new immune classification. Type AI: inflammatory cells negative and both negative (CMTM6Low/PD-L1−); type AII: inflammatory cells negative and single positive (CMTM6High/PD-L1− or CMTM6Low/PD-L1+); type AIII: inflammatory cells negative and both positive (CMTM6High/PD-L1+). **f**, **g** Dot plots of inflammatory cell density in different subgroups of MTM type and non-MTM type. Quantitative data are presented as mean ± SD. *PD-L1−* PD-L1-negative, *PD-L1+* PD-L1-positive (JPG 1251 kb)

Supplementary file2. Kaplan–Meier survival curves for DFS of HCC patients according to CMTM6 and PD-L1 expression. **a** DFS according to CMTM6 expression status in the whole population, MTM and non-MTM type HCC. **b** DFS according to tumoral PD-L1 expression status in the whole population, MTM and non-MTM type HCC. **c** DFS according to a combination of CMTM6/PDL1 coexpression. Group I: CMTM6Low/PD-L1−; Group II: CMTM6High/PD-L1− or CMTM6Low/PD-L1+; Group III: CMTM6High/PD-L1+. **d** DFS according to new immune classification. Type BI: inflammatory cells positive and both negative (CMTM6Low/PD-L1−); type BII: inflammatory cells positive and single positive (CMTM6High/PD-L1− or CMTM6Low/PD-L1+); type BIII: inflammatory cells positive and both positive (CMTM6High/PD-L1+). **e** DFS according to new immune classification. Type AI: inflammatory cells negative and both negative (CMTM6Low/PD-L1−); type AII: inflammatory cells negative and single positive (CMTM6High/PD-L1− or CMTM6Low/PD-L1+); type AIII: inflammatory cells negative and both positive (CMTM6High/PD-L1+). *PD-L1−* PD-L1-negative, *PD-L1+* PD-L1-positive (JPG 933 kb)

Supplementary file3. Stratified analysis of CMTM6 expression related to OS. The correlation of CMTM6 expression and OS in the indicated groups (JPG 557 kb)

Supplementary file4 (DOCX 51 kb)
