# Supplementary figures and images for: Coexpression of CMTM6 and PD-L1 as a predictor of poor prognosis in macrotrabecular-massive hepatocellular carcinoma
Source: Cancer Immunol Immunother. 2020 Aug 7;70(2):417–29. doi: 10.1007/s00262-020-02691-9 (PMC7889680; doi:10.1007/s00262-020-02691-9)

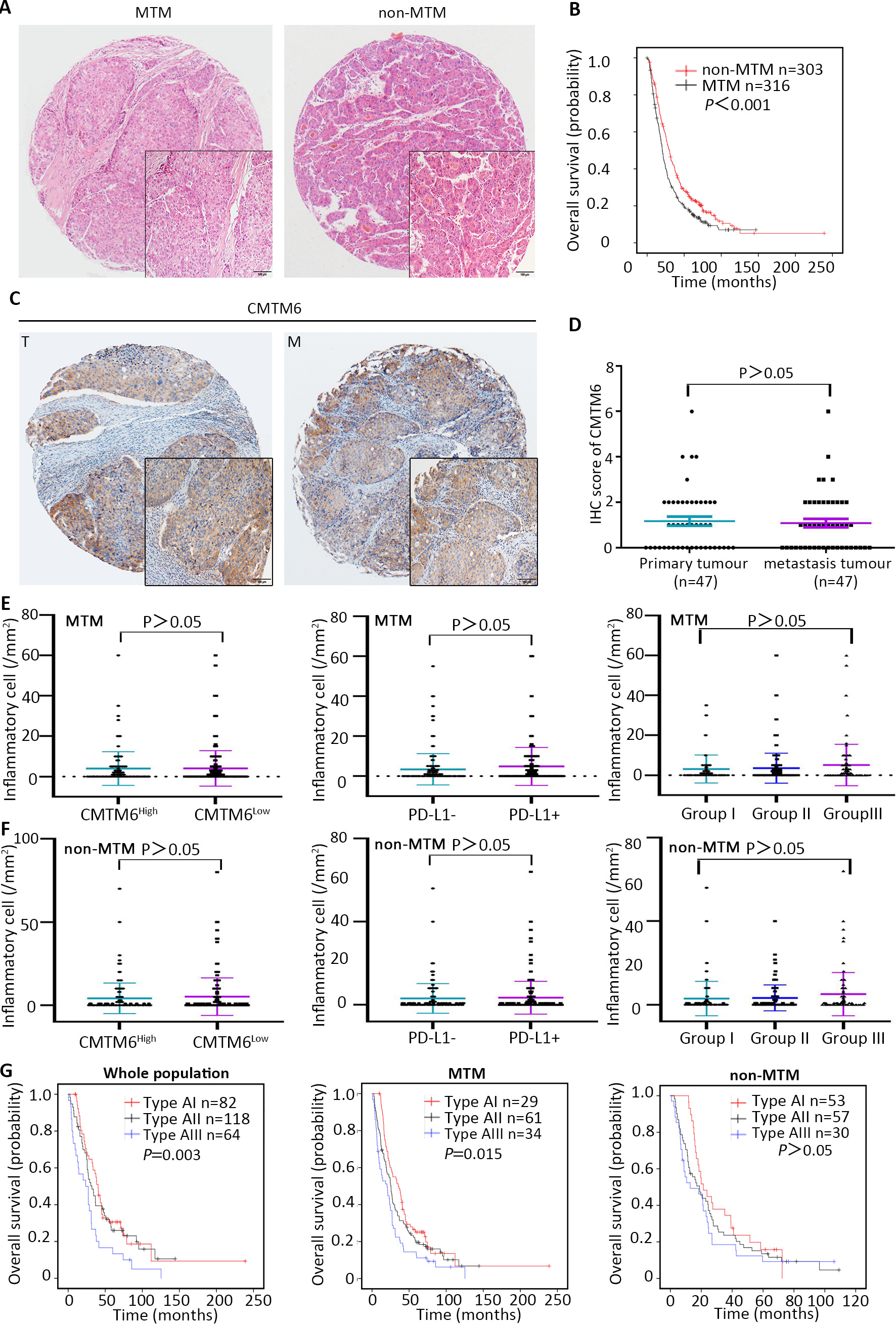

Supplement: Supplementary file 1 — Supplementary file1. a Representative photomicrographs are shown for the MTM type and non-MTM type. b Kaplan–Meier survival curves for OS in MTM type vs. non-MTM type tumor. c CMTM6 expression in 47 HCC metastasis cases analyzed by IHC. Representative photomicrographs are shown for the primary tumor (T) and metastatic (M) lesions. d Comparison of CMTM6 levels between the primary tumor and metastatic lesions. e OS according to new immune classification. Type AI: inflammatory cells negative and both negative (CMTM6Low/PD-L1−); type AII: inflammatory cells negative and single positive (CMTM6High/PD-L1− or CMTM6Low/PD-L1+); type AIII: inflammatory cells negative and both positive (CMTM6High/PD-L1+). f, g Dot plots of inflammatory cell density in different subgroups of MTM type and non-MTM type. Quantitative data are presented as mean ± SD. PD-L1− PD-L1-negative, PD-L1+ PD-L1-positive (JPG 1251 kb) [file 262_2020_2691_MOESM1_ESM.jpg]

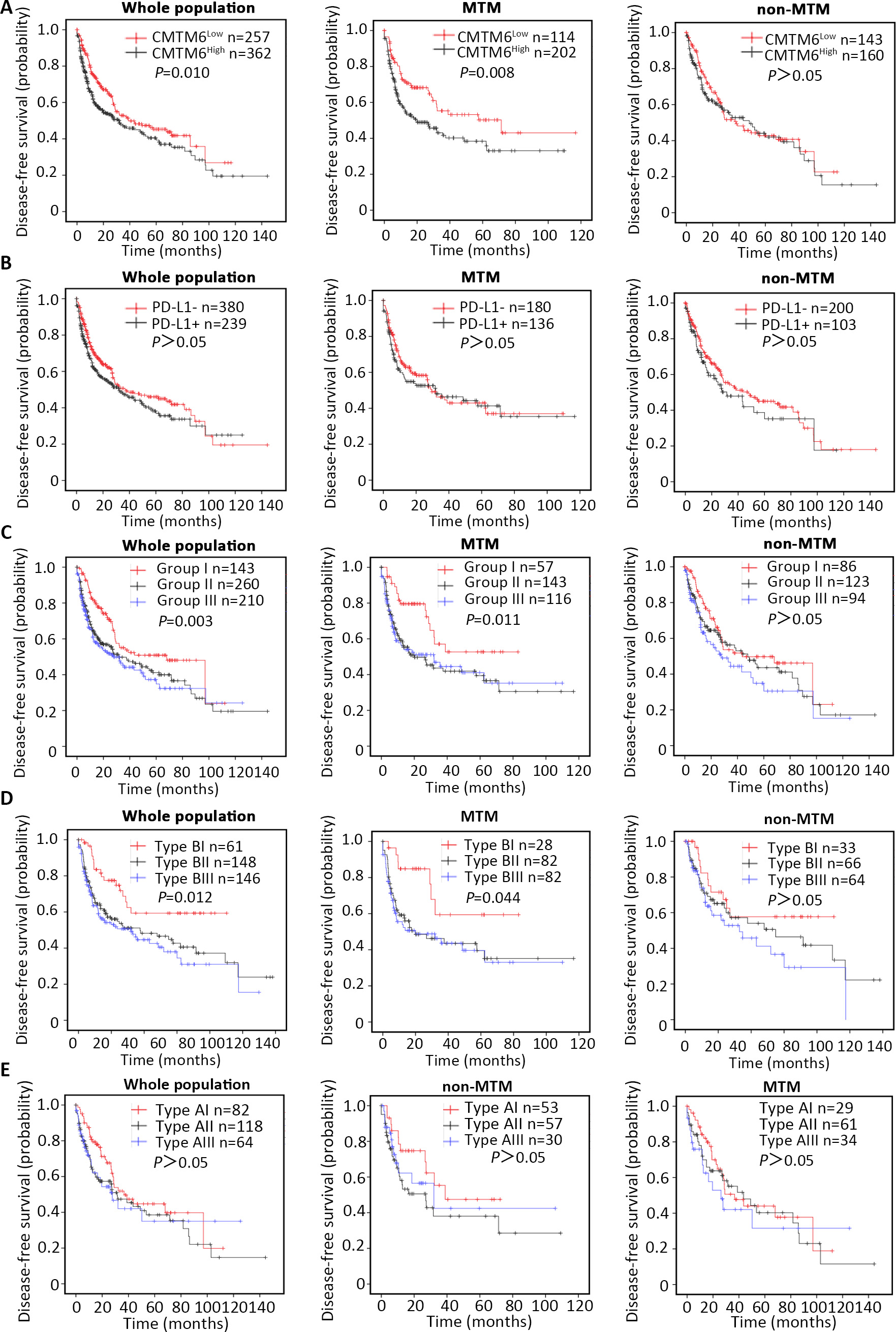

Supplement: Supplementary file 2 — Supplementary file2. Kaplan–Meier survival curves for DFS of HCC patients according to CMTM6 and PD-L1 expression. a DFS according to CMTM6 expression status in the whole population, MTM and non-MTM type HCC. b DFS according to tumoral PD-L1 expression status in the whole population, MTM and non-MTM type HCC. c DFS according to a combination of CMTM6/PDL1 coexpression. Group I: CMTM6Low/PD-L1−; Group II: CMTM6High/PD-L1− or CMTM6Low/PD-L1+; Group III: CMTM6High/PD-L1+. d DFS according to new immune classification. Type BI: inflammatory cells positive and both negative (CMTM6Low/PD-L1−); type BII: inflammatory cells positive and single positive (CMTM6High/PD-L1− or CMTM6Low/PD-L1+); type BIII: inflammatory cells positive and both positive (CMTM6High/PD-L1+). e DFS according to new immune classification. Type AI: inflammatory cells negative and both negative (CMTM6Low/PD-L1−); type AII: inflammatory cells negative and single positive (CMTM6High/PD-L1− or CMTM6Low/PD-L1+); type AIII: inflammatory cells negative and both positive (CMTM6High/PD-L1+). PD-L1− PD-L1-negative, PD-L1+ PD-L1-positive (JPG 933 kb) [file 262_2020_2691_MOESM2_ESM.jpg]

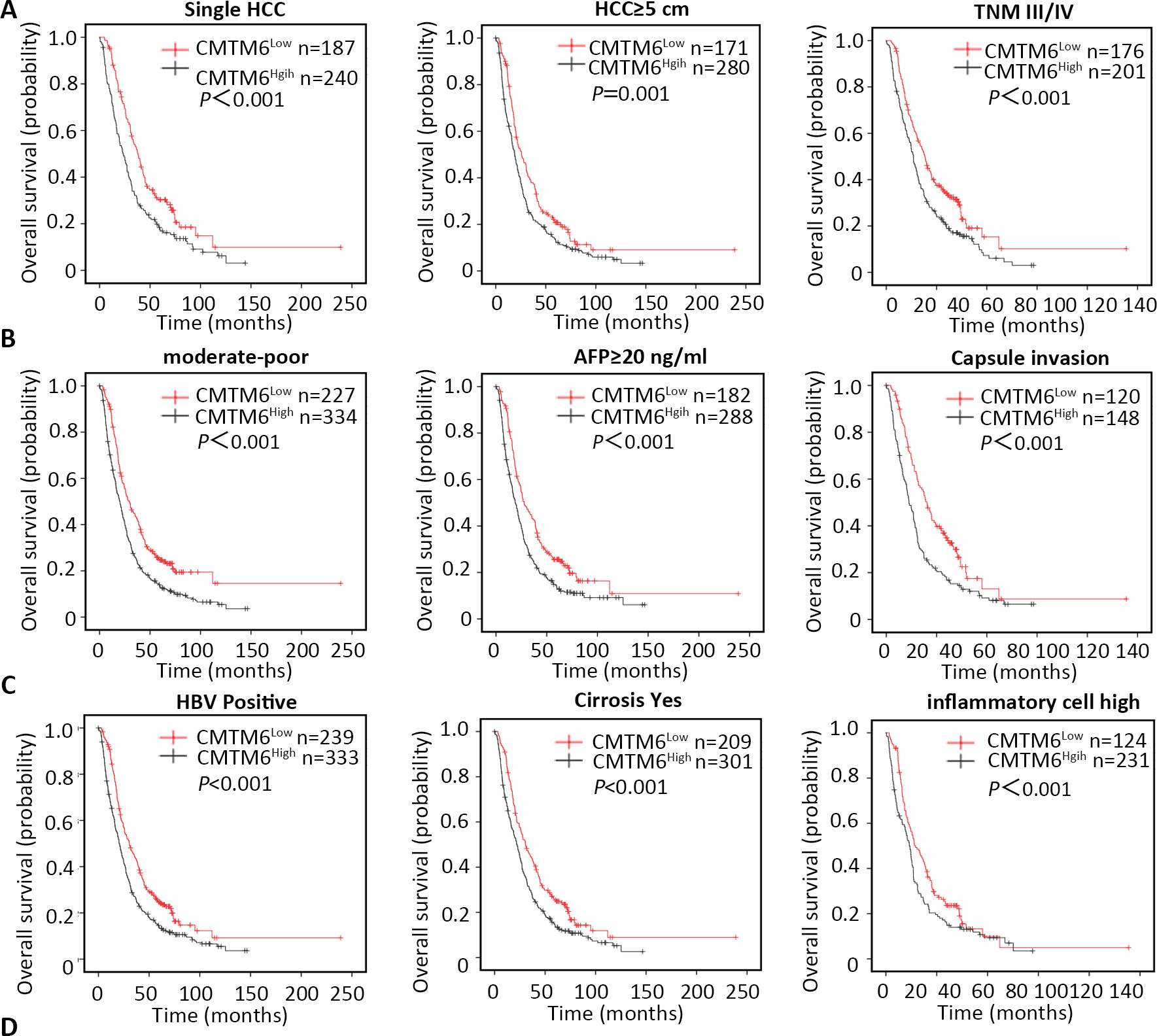

Supplement: Supplementary file 3 — Supplementary file3. Stratified analysis of CMTM6 expression related to OS. The correlation of CMTM6 expression and OS in the indicated groups (JPG 557 kb) [file 262_2020_2691_MOESM3_ESM.jpg]
