# Supplementary material for: Coexpression of CMTM6 and PD-L1 as a predictor of poor prognosis in macrotrabecular-massive hepatocellular carcinoma
Source: Cancer Immunol Immunother. 2020 Aug 7;70(2):417–29. doi: 10.1007/s00262-020-02691-9 (PMC7889680; doi:10.1007/s00262-020-02691-9)
Supplement: Supplementary file 4 — Supplementary file4 (DOCX 51 kb) [file 262_2020_2691_MOESM4_ESM.docx]

**Supplementary Table 1. Baseline characteristics of the patients according to histological subtype.**

|  | **Whole population** | **MTM** | **Non-MTM** | ***P* value** |
| --- | --- | --- | --- | --- |
| Age | 50±12 | 47±11.8 | 52±12.0 | 0.450 |
| Gender |  |  |  | 0.065 |
| Male | 549(88.7%) | 273(86.4%) | 276(91.1%) |  |
| Female | 70(11.3%) | 43(13.6%) | 27(8.9%) |  |
| HBV |  |  |  | 0.998 |
| Positive | 47(7.6%) | 24(7.6%) | 23(7.6%) |  |
| Negative | 572(92.4%) | 292(92.4%) | 280(92.4%) |  |
| AFP (ng/ml) |  |  |  | **0.005** |
| <20 | 149(24.1%) | 61(19.3%) | 88(29.0%) |  |
| ≥20 | 470(75.9%) | 255(80.7%) | 215(71.0%) |  |
| Cirrhosis |  |  |  | 0.442 |
| Yes | 109(17.6%) | 52(16.5%) | 57(18.8%) |  |
| No | 510(82.4%) | 264(83.5%) | 246(81.2%) |  |
| Tumor multiplicity |  |  |  | 0.165 |
| Single | 427(69%) | 210(66.5%) | 217(71.6%) |  |
| Multiple | 192(31%) | 106(33.5%) | 86(28.4%) |  |
| Tumor size^b^ |  |  |  | **0.004** |
| <5 cm | 168(27.1%) | 70(22.2%) | 98(32.3%) |  |
| ≥5 cm | 451(72.9%) | 246(77.8%) | 205(67.7%) |  |
| Differentiation |  |  |  | **0.018** |
| Well | 58(9.4%) | 21(6.6%) | 37(12.2%) |  |
| Moderate-Poor | 561(90.6%) | 295(93.4%) | 266(87.8%) |  |
| TNM Stage |  |  |  | **0.027** |
| I-II | 377(60.9%) | 179(56.5%) | 198(65.3%) |  |
| III-IV | 242(39.1%) | 137(43.4%) | 105(34.7%) |  |
| Vascular invasion |  |  |  | **0.000** |
| No | 521(84.2%) | 250(79.1%) | 271(89.4%) |  |
| Yes | 98(15.8%) | 66(20.9%) | 32(10.6%) |  |
| Tumor capsule |  |  |  | 0.079 |
| Complete | 351(56.7%) | 190(60.1%) | 161(53.1%) |  |
| Incomplete | 268(43.3%) | 126(39.9%) | 142(48.9%) |  |
| LN metastasis |  |  |  | 0.809 |
| No | 587(94.8%) | 299(94.65) | 288(95%) |  |
| Yes | 32(5.2%) | 17(5.4%) | 15(5%) |  |
| Cytological type |  |  |  | 0.825 |
| Liver cell | 538(86.9%) | 277(87.7%) | 261(86.1%) |  |
| Clear cell | 40(6.5%) | 19(6.1%) | 21(6.8%) |  |
| Fatty-rich | 33(5.3%) | 16(5.1%) | 17(5.6%) |  |
| Giant cell | 8(1.3%) | 4(1.3%) | 4(1.3%) |  |
| CMTM6 |  |  |  | **0.005** |
| Low | 257(41.5%) | 114(36.1%) | 243(47.2%) |  |
| High | 362(58.5%) | 202(63.9%) | 160(52.8%) |  |
| Tumoral PD-L1 |  |  |  | **0.021** |
| Negative | 380(61.4%) | 180(57.0%) | 200(66.0%) |  |
| positive | 239(38.6%) | 136(43.0%) | 103(34.0%) |  |
| Inflammatory cell PD-L1 |  |  |  | 0.356 |
| Negative | 309(49.9%) | 152(48.1%) | 157(51.8%) |  |
| positive | 310(50.1%) | 164(51.9%) | 146(48.2%) |  |
| Inflammatory cells |  |  |  | 0.080 |
| low | 264(42.6%) | 124(39.2%) | 140(46.2%) |  |
| high | 355(57.4%) | 192(60.8%) | 163(53.8%) |  |
| Group |  |  |  | **0.009** |
| I | 143(23.1%) | 57(18.0%) | 86(28.4%) |  |
| II | 266(43%) | 143(45.3%) | 123(40.6%) |  |
| III | 210(33.9%) | 116(36.7%) | 94(31.0%) |  |
| Type |  |  |  | **0.042** |
| AI | 82(13.2%) | 29(9.2%) | 53(17.5%) |  |
| AII | 118(19.1%) | 61(19.3%) | 57(18.8%) |  |
| AIII | 64(10.3%) | 34(10.8%) | 30(9.9%) |  |
| BI | 61(9.9%) | 28(8.9%) | 33(10.9%) |  |
| BII | 148(23.9%) | 82(25.9%) | 66(21.8%) |  |
| BIII | 146(23.6%) | 82(25.9%) | 64(21.1%) |  |

*P value＜0.05 in bold are statistically significant. AFP, a-fetoprotein; HBV, hepatitis B virus infection; LN, lymph node. aMedian age; bMedian tumor size.

**Supplementary Table 2. The association of clinicopathological parameters with tumoral PD-L1 expression in the MTM and non-MTM HCC.**

|  | **Whole population (n=619)** | | | |  | **MTM (n=316)** | | | |  | **Non-MTM (n=303)** | | | |
| --- | --- | --- | --- | --- | --- | --- | --- | --- | --- | --- | --- | --- | --- | --- |
|  |  | **Tumoral PD-L1 expression** | | |  |  | **Tumoral PD-L1 expression** | | |  |  | **Tumoral PD-L1 expression** | | |
| **Variables** | **n** | **-** | **+** | **P value** |  | **n** | **-** | **+** | **P value** |  | **n** | **-** | **+** | **P value** |
| Age (y) ^a^ |  |  |  | **0.027** |  |  |  |  | **0.032** |  |  |  |  | 0.726 |
| ＜50 | 291 | 192 | 99 |  |  | 166 | 104 | 62 |  |  | 276 | 183 | 93 |  |
| ≥50 | 328 | 188 | 140 |  |  | 150 | 76 | 74 |  |  | 27 | 17 | 10 |  |
| Gender |  |  |  | 0.789 |  |  |  |  | 0.406 |  |  |  |  | 0.176 |
| Male | 549 | 336 | 213 |  |  | 273 | 153 | 120 |  |  | 125 | 88 | 37 |  |
| Female | 70 | 44 | 26 |  |  | 43 | 27 | 16 |  |  | 178 | 112 | 66 |  |
| HBV |  |  |  | 0.130 |  |  |  |  | **0.045** |  |  |  |  | 0.934 |
| Positive | 47 | 24 | 23 |  |  | 24 | 9 | 15 |  |  | 23 | 15 | 8 |  |
| Negative | 572 | 356 | 216 |  |  | 292 | 171 | 121 |  |  | 280 | 185 | 95 |  |
| AFP (ng/ml) |  |  |  | **0.016** |  |  |  |  | **0.008** |  |  |  |  | 0.609 |
| ＜20 | 149 | 104 | 45 |  |  | 61 | 44 | 17 |  |  | 88 | 60 | 28 |  |
| ≥20 | 470 | 276 | 194 |  |  | 255 | 136 | 119 |  |  | 215 | 140 | 75 |  |
| Cirrhosis |  |  |  | 0.049 |  |  |  |  | 0.672 |  |  |  |  | **0.022** |
| Yes | 109 | 76 | 33 |  |  | 52 | 31 | 21 |  |  | 57 | 45 | 12 |  |
| No | 510 | 304 | 206 |  |  | 264 | 149 | 115 | 0.740 |  | 246 | 155 | 91 |  |
| Tumor multiplicity |  |  |  | 0.461 |  | 210 | 121 | 89 |  |  |  |  |  | 0.094 |
| Single | 427 | 258 | 169 |  |  | 106 | 59 | 47 |  |  | 217 | 137 | 80 |  |
| Multiple | 192 | 122 | 70 |  |  |  |  |  |  |  | 86 | 63 | 23 |  |
| Tumor size^b^ |  |  |  | 0.561 |  |  |  |  | 0.108 |  |  |  |  | 0.733 |
| ＜5 cm | 168 | 100 | 68 |  |  | 70 | 34 | 36 |  |  | 98 | 66 | 32 |  |
| ≥5 cm | 451 | 280 | 171 |  |  | 246 | 146 | 100 |  |  | 205 | 134 | 71 |  |
| Differentiation |  |  |  | **0.036** |  |  |  |  | 0.353 |  |  |  |  | 0.090 |
| Well | 58 | 43 | 15 |  |  | 21 | 14 | 7 |  |  | 37 | 29 | 8 |  |
| Moderate-Poor | 561 | 337 | 224 |  |  | 295 | 166 | 129 |  |  | 266 | 171 | 95 |  |
| TNM Stage |  |  |  | 0.808 |  |  |  |  | 0..653 |  |  |  |  | 0.860 |
| I-II | 377 | 230 | 147 |  |  | 179 | 100 | 79 |  |  | 198 | 130 | 68 |  |
| III-IV | 242 | 150 | 92 |  |  | 137 | 80 | 57 |  |  | 105 | 70 | 35 |  |
| Vascular invasion |  |  |  | 0.347 |  |  |  |  | 0.468 |  |  |  |  | 0.962 |
| No | 521 | 324 | 197 |  |  | 250 | 145 | 105 |  |  | 271 | 179 | 92 |  |
| Yes | 98 | 56 | 42 |  |  | 66 | 35 | 31 |  |  | 32 | 21 | 11 |  |
| Tumor capsule |  |  |  | 0.456 |  |  |  |  | 0.681 |  |  |  |  | 0.200 |
| Complete | 351 | 211 | 140 |  |  | 190 | 110 | 80 |  |  | 161 | 101 | 60 |  |
| Incomplete | 268 | 169 | 99 |  |  | 126 | 70 | 56 |  |  | 142 | 99 | 43 |  |
| LN metastasis |  |  |  | 0.810 |  |  |  |  | 0.873 |  |  |  |  | 0.614 |
| No | 587 | 361 | 226 |  |  | 299 | 170 | 129 |  |  | 288 | 191 | 97 |  |
| Yes | 32 | 19 | 13 |  |  | 17 | 10 | 7 |  |  | 15 | 9 | 6 |  |
| Cytological type |  |  |  | 0.761 |  |  |  |  | 0.679 |  |  |  |  | 0.076 |
| Liver cell | 538 | 326 | 212 |  |  | 277 | 161 | 116 |  |  | 261 | 165 | 96 |  |
| Clear cell | 40 | 27 | 13 |  |  | 19 | 10 | 9 |  |  | 21 | 17 | 4 |  |
| Fatty-rich | 33 | 22 | 11 |  |  | 16 | 7 | 9 |  |  | 17 | 15 | 2 |  |
| Giant cell | 8 | 5 | 3 |  |  | 4 | 2 | 2 |  |  | 4 | 3 | 1 |  |
| Inflammatory cell PD-L1 |  |  |  | **0.000** |  |  |  |  | **0.029** |  |  |  |  | **0.000** |
| Negative | 264 | 193 | 71 |  |  | 124 | 80 | 44 |  |  | 140 | 113 | 277 |  |
| Positive | 355 | 187 | 168 |  |  | 192 | 100 | 92 |  |  | 163 | 87 | 76 |  |
| Inflammatory cells |  |  |  | **0.000** |  |  |  |  | **0.000** |  |  |  |  | **0.000** |
| Low | 309 | 285 | 24 |  |  | 152 | 139 | 13 |  |  | 157 | 146 | 11 |  |
| High | 310 | 95 | 215 |  |  | 164 | 41 | 123 |  |  | 146 | 54 | 92 |  |

*P value＜0.05 in bold are statistically significant. AFP, a-fetoprotein; HBV, hepatitis B virus infection; LN, lymph node. aMedian age; bMedian tumor size.

**Supplementary Table 3. Univariate analyses of prognostic factors correlated with DFS in MTM and non-MTM HCC.**

| **Variables** | **Whole population** | |  | **MTM** | |  | **Non-MTM** | |
| --- | --- | --- | --- | --- | --- | --- | --- | --- |
|  | **HR (95% CI)** | ***P* value** |  | **HR (95% CI)** | ***P* value** |  | **HR (95% CI)** | ***P* value** |
| **Disease-free survival** |  |  |  |  |  |  |  |  |
| Age (y) (<50 vs. ≥50 years) | 0.838(0.664-1.058) | 0.138 |  | 0.847(0.516-1.388) | 0.509 |  | 0.866(0.620-1.211) | 0.401 |
| Gender (Male vs. Female) | 0.693(0.463-1.038) | 0.076 |  | 0.841(0.605-1.170) | 0.305 |  | 0.460(0.225-0.941) | **0.033** |
| HBV (Positive vs. Negative) | 1.060(0.691-1.625) | 0.789 |  | 1.227(0.662-2.273) | 0.515 |  | 0.951(0.525-1.722) | 0.868 |
| AFP (ng/ml) (＜20 vs. ≥20) | 1.183(0.902-1.552) | 0.225 |  | 1.374(0.879-2.147) | 0.163 |  | 0.987(0.691-1.409) | 0.942 |
| Cirrhosis (No vs. Yes) | 1.053(0.774-1.432) | 0.744 |  | 0.984(0.635-1.526) | 0.943 |  | 1.117(0.724-1.722) | 0.617 |
| Tumor multiplicity (Single vs. Multiple) | 1.210(0937-1.564) | 0.144 |  | 1.163(0.820-1.651) | 0.396 |  | 1.251(0.856-1.827) | 0.247 |
| Tumor size (cm) (＜5 vs. ≥5) | 1.175(0.909-1.519) | 0.217 |  | 1.168(0.793-1.720) | 0.433 |  | 1.129(0.797-1.599) | 0.494 |
| Differentiation (Well vs. Moderate-Poor) | 1.191(0.820-1.731) | 0.359 |  | 1.530(0.779-3.008) | 0.217 |  | 1.017(0.644-1.606) | 0.942 |
| TNM Stage (I-II vs. III-IV) | 1.051(0.822-1.345) | 0.691 |  | 1.045(0.746-1.462) | 0.799 |  | 1.033(0.717-1.490) | 0.860 |
| Vascular invasion (No vs. Yes) | 1.295(0.924-1.815) | 0.133 |  | 1.070(0.703-1.629) | 0.752 |  | 1.676(0.938-2.996) | 0.081 |
| Tumor capsule (Complete vs. Incomplete) | 0.928(0.734-1.173) | 0.534 |  | 0.888(0.635-1.241) | 0.485 |  | 0.988(0.709-1.378) | 0.944 |
| LN metastasis (No vs. Yes) | 0.891(0.458-1.734) | 0.734 |  | 0.920(0.406-2.084) | 0.841 |  | 0.863(0.273-2.727) | 0.801 |
| Cytological type (Liver cell vs. Clear cell vs. Fatty-rich vs. Giant cell) | 0.880(0.715-1.082) | 0.225 |  | 0.926(0.699-1.227) | 0.593 |  | 0.847(0.625-1.148) | 0.285 |
| Tumoral PD-L1 (- vs. +) | 1.163(0.915-1.476) | 0.217 |  | 1.222(0.858-1.741) | 0.267 |  | 1.126(0.809-1.566) | 0.483 |
| Inflammatory cell PD-L1 (- vs. +) | 1.252(0.992-1.581) | 0.058 |  | 1.182(1.643-1.735) | 0.321 |  | 1.379(0.990-1.922) | 0.058 |
| Inflammatory cells (low vs. high) | 1.025(0.810-1.296) | 0.839 |  | 1.100(0.782-1.547) | 0.583 |  | 0.950(0.682-1.323) | 0.762 |
| CMTM6 expression (low vs. high) | 1.369(1.078-1.739) | **0.010** |  | 1.624(1.135-2.324) | **0.008** |  | 1.125(0.807-1.568) | 0.488 |

*P value＜0.05 in bold are statistically significant. AFP, a-fetoprotein; HBV, hepatitis B virus infection; LN, lymph node. aMedian age; bMedian tumor size.

**Supplementary Table 4. Multivariate analyses of prognostic factors correlated with OS in MTM and non-MTM HCC.**

| **Variables** | | **Whole population** | |  | **MTM** | |  | | **Non-MTM** | | |
| --- | --- | --- | --- | --- | --- | --- | --- | --- | --- | --- | --- |
|  | **HR (95% CI)** | ***P* value** |  | **HR (95% CI)** | ***P* value** |  | **HR (95% CI)** | | ***P* value** |  |  |
| **Overall survival** | |  |  |  |  |  |  | |  |  | |
| Gender (Male vs. Female) | |  |  |  | 0.620(0.385-0.999) | **0.049** |  | |  |  | |
| AFP (ng/ml) (＜20 vs. ≥20) | | 1.021(0.830-1.257) | 0.842 |  |  |  |  | |  |  | |
| Tumor multiplicity (Single vs. Multiple) | | 1.362(1.111-1.670) | **0.003** |  | 1.624(1.196-2.205) | **0.002** |  | | 1.181(0.893-1.560) | 0.243 | |
| Tumor size (cm) (＜5 vs. ≥5) | | 1.418(1.131-1.778) | **0.002** |  | 1.394(0.998-1.947) | 0.052 |  | | 1.461(1.052-2.028) | **0.024** | |
| Differentiation (Well vs. Moderate-Poor) | | 1.279(0.942-1.737) | 0.115 |  | 1.350(0.905-2.016) | 0.142 |  | |  |  | |
| TNM Stage (I-II vs. III-IV) | | 1.111(0.886-1.394) | 0.363 |  | 1.062(0.745-1.514) | 0.740 |  | | 1.159(0.858-1.567) | 0.337 | |
| Vascular invasion (No vs. Yes) | | 1.730(1.338-2.237) | **0.000** |  | 2.213(1.428-3.430) | **0.000** |  | | 1.581(1.157-2.160) | **0.004** | |
| Tumor capsule (Complete vs. Incomplete) | | 0.911(0.755-1.098) | 0.327 |  | 0.782(0.595-1.027) | 0.077 |  | |  |  | |
| LN metastasis (No vs. Yes) | | 2.045(1.394-2.998) | **0.000** |  | 3.757(2.165-6.520) | **0.000** |  | | 1.409(0.823-2.411) | 0.211 | |
| Tumoral PD-L1 (- vs. +) | | 1.037(0.841-1.278) | 0.734 |  |  |  |  | |  |  | |
| CMTM6/PD-L1 group I/II/III | | 1.213(1.059-1.389) | **0.005** |  | 1.297(1.094-1.536) | **0.003** |  | | 1.115(0.950-1.309) | 0.183 | |

*P value＜0.05 in bold are statistically significant. AFP, a-fetoprotein; HBV, hepatitis B virus infection; LN, lymph node; HR, hazard ratio; CI, confidence interval. ^a^Median age; ^b^Median tumor size.
